# Cancer causes emergency home visits

**DOI:** 10.1002/jgf2.472

**Published:** 2021-06-21

**Authors:** Koki Kato, Masaya Tomita, Moe Kato, Takaaki Goto, Kyukei Nishizono

**Affiliations:** ^1^ Madoka Family Clinic Fukuoka Japan; ^2^ Hokkaido Centre for Family Medicine Academic and Research Centre Hokkaido Japan; ^3^ Ebetsu Home Care Clinic Hokkaido Japan; ^4^ Sakuragaoka Clinic Hokkaido Japan; ^5^ Naito Clinic Miyagi Japan; ^6^ Iizuka Memorial Hospital Fukuoka Japan

## CONFLICT OF INTEREST

The other authors have stated explicitly that there are no conflicts of interest in connection with this article.

To the Editor,

In their recently published study, Kuroda et al.^1^ investigated the factors associated with emergency home visits (EHVs). They analyzed a total of 608 EHVs in 214 patients within the 2‐year duration and reported that higher care‐needed level, urinary catheter use, and central venous port use showed a positive correlation with the frequency of EHVs. On the other hand, the association between the cancer‐bearing state and the frequency of EHVs was not statistically significant. However, Kato et al.^2^ analyzed 278 EHVs and demonstrated different results from the study; the incidence rate ratio of the cancer‐bearing state was 4.71 (95% confidence interval, 2.60–8.52). Why do the two results differ?

Kuroda et al. did not show summary statistics of the duration of the follow‐up period of each case. Those periods are vital information for the analyses. For example, the statistical weight is more massive in a count of 10 EHVs out of 14 days than the same count out of 730 days. Cancer patients usually have a shorter follow‐up period because their prognoses are worse than noncancer patients. Kato et al.^2^ reported that the median follow‐up period was 42 days in cancer patients and 82 days in noncancer patients out of the 3‐month study period. The shorter follow‐up of cancer patients was caused by death at home or hospitalization. Although Kuroda et al. did not mention whether they included the time information in the Poisson and negative binomial regressions, they should have incorporated an offset variable^3^ in the models if they did not. The lack of the information could cause an underestimation of the weighing of the cancer‐bearing state variable for EHVs.

In conclusion, this study is informative because it demonstrated the factors of emergency home visits. However, we think they need to implement the duration of the follow‐up period for each case into their analyses to determine the appropriate statistical weight of each factor.
